# Current cell therapies for systemic lupus erythematosus

**DOI:** 10.1093/stcltm/szae044

**Published:** 2024-06-26

**Authors:** Lan T M Dao, Thu Thuy Vu, Quyen Thi Nguyen, Van T Hoang, Thanh Liem Nguyen

**Affiliations:** Vinmec Research Institute of Stem Cell and Gene Technology, Vinmec Healthcare System, Hanoi 100000, Vietnam; Vinmec Research Institute of Stem Cell and Gene Technology, Vinmec Healthcare System, Hanoi 100000, Vietnam; Vinmec Research Institute of Stem Cell and Gene Technology, Vinmec Healthcare System, Hanoi 100000, Vietnam; Vinmec Research Institute of Stem Cell and Gene Technology, Vinmec Healthcare System, Hanoi 100000, Vietnam; Vinmec Research Institute of Stem Cell and Gene Technology, Vinmec Healthcare System, Hanoi 100000, Vietnam; Vinmec International Hospital, Center of Regenerative Medicine and Cell Therapy, Vinmec Healthcare System, Hanoi 100000, Vietnam; Vin University, College of Health Sciences, Hanoi 100000, Vietnam

**Keywords:** systemic lupus erythematosus, cell therapy, clinical trial, hematopoietic stem cell transplantation, mesenchymal stem cell therapy, chimeric antigen receptor T-cell therapy

## Abstract

Systemic lupus erythematosus (SLE) is a chronic autoimmune disease in which multiple organs are damaged by the immune system. Although standard treatment options such as hydroxychloroquine (HCQ), glucocorticoids (GCs), and other immunosuppressive or immune-modulating agents can help to manage symptoms, they do not offer a cure. Hence, there is an urgent need for the development of novel drugs and therapies. In recent decades, cell therapies have been used for the treatment of SLE with encouraging results. Hematopoietic stem cell transplantation, mesenchymal stem cells, regulatory T (Treg) cell, natural killer cells, and chimeric antigen receptor T (CAR T) cells are advanced cell therapies which have been developed and evaluated in clinical trials in humans. In clinical application, each of these approaches has shown advantages and disadvantages. In addition, further studies are necessary to conclusively establish the safety and efficacy of these therapies. This review provides a summary of recent clinical trials investigating cell therapies for SLE treatment, along with a discussion on the potential of other cell-based therapies. The factors influencing the selection of common cell therapies for individual patients are also highlighted.

## Graphical Abstract

**Graphical Abstract:**
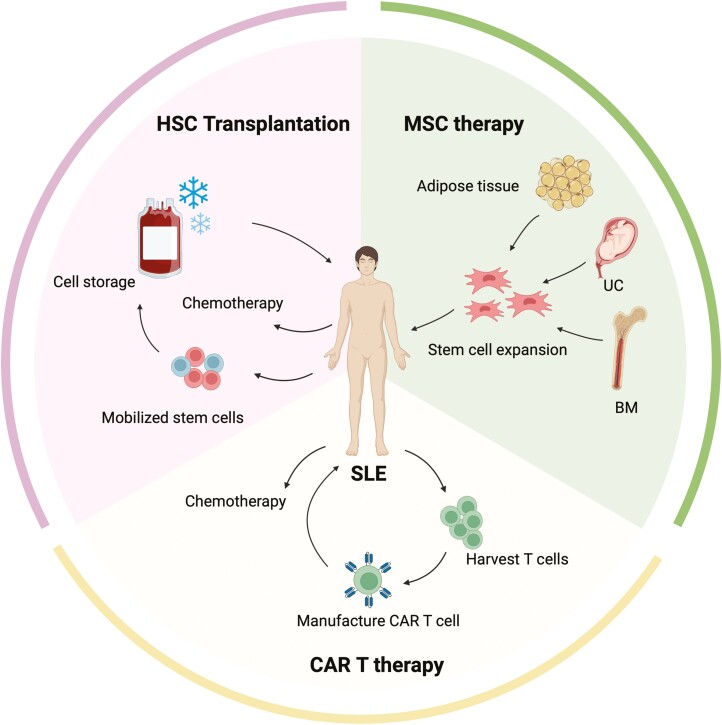

**Significance statement**

Cell therapy is a promising treatment option for SLE, particularly for individuals who are refractory to conventional therapies. Herein, we summarize the current knowledge of cell-based therapies for the treatment of SLE, offering insights into the outcomes of the most recent human clinical trials. We also discuss the advantages and disadvantages of current cell therapies and highlight the factors that may affect the selection of common cell therapies for an individual patient. In addition, we provide an update on the potential of other cell-based therapies for the management of SLE.

## Introduction

Systemic lupus erythematosus (SLE) is a complex, chronic autoimmune disease involving various organs and systems. In patients with SLE, the immune system malfunctions and produces autoantibodies that attack the body’s own tissues.^1^ At least 5 million people worldwide are affected by a form of lupus. The incidence is the highest among women of reproductive age.^1,2^ The symptoms of SLE may vary among patients. Fatigue and joint pain are the most prevalent symptoms, followed by photosensitivity, myalgia, rash, and fever.^3^ A combination of genetic and environmental factors is believed to cause this disease.^4,5^

To date, there is no cure for SLE. Generally, patients are prescribed anti-inflammatory medications, corticosteroids, immunosuppressants, and other biologics. However, these medications may exhibit significant side effects, and there is still a high number of refractory patients.^1,6^ Consequently, the quest for more effective treatments has become a foremost concern.

Cell therapy involves the transplantation of either autologous or allogeneic cellular material to a patient for medical purposes. In recent decades, both preclinical investigations and clinical trials have shown promising results from cell-based therapies for the treatment of SLE. The most extensively cell therapies studied for SLE are hematopoietic stem cell transplantation (HSCT), mesenchymal stem cell (MSC) therapy, and, more recently, chimeric antigen receptor (CAR) T-cell therapy. HSCT was the first-cell therapy implemented for patients with SLE. However, until now, only a limited number of patients have been treated.^7^ MSC therapy has been extensively studied and more widely implemented in human trials. Recently, CAR T-cell therapy was successfully administered to patients with refractory SLE using CD19-targeted T cells.^8^ Additionally, regulatory T (Treg) and natural killer (NK) cell therapies for SLE have been explored, although research data and clinical experience are still limited. Overall, cell-based therapies for SLE have been well tolerated and shown to be safe, but evidence of their beneficial effects is controversial. Hence, further studies evaluating the long-term safety and efficacy of these advanced treatment options are ongoing.

In this review, we focus on the current understanding of various clinical studies of HSCT and MSCs, and CAR T cells, derived from the latest outcomes of human trials. We denote the advantages and disadvantages of different approaches and highlight the factors that may affect the choice of cell therapies for a given patient. In addition, other preclinical cell therapies are discussed to assess their potential as alternative treatments.

## Challenges in SLE management

**Figure 1.**
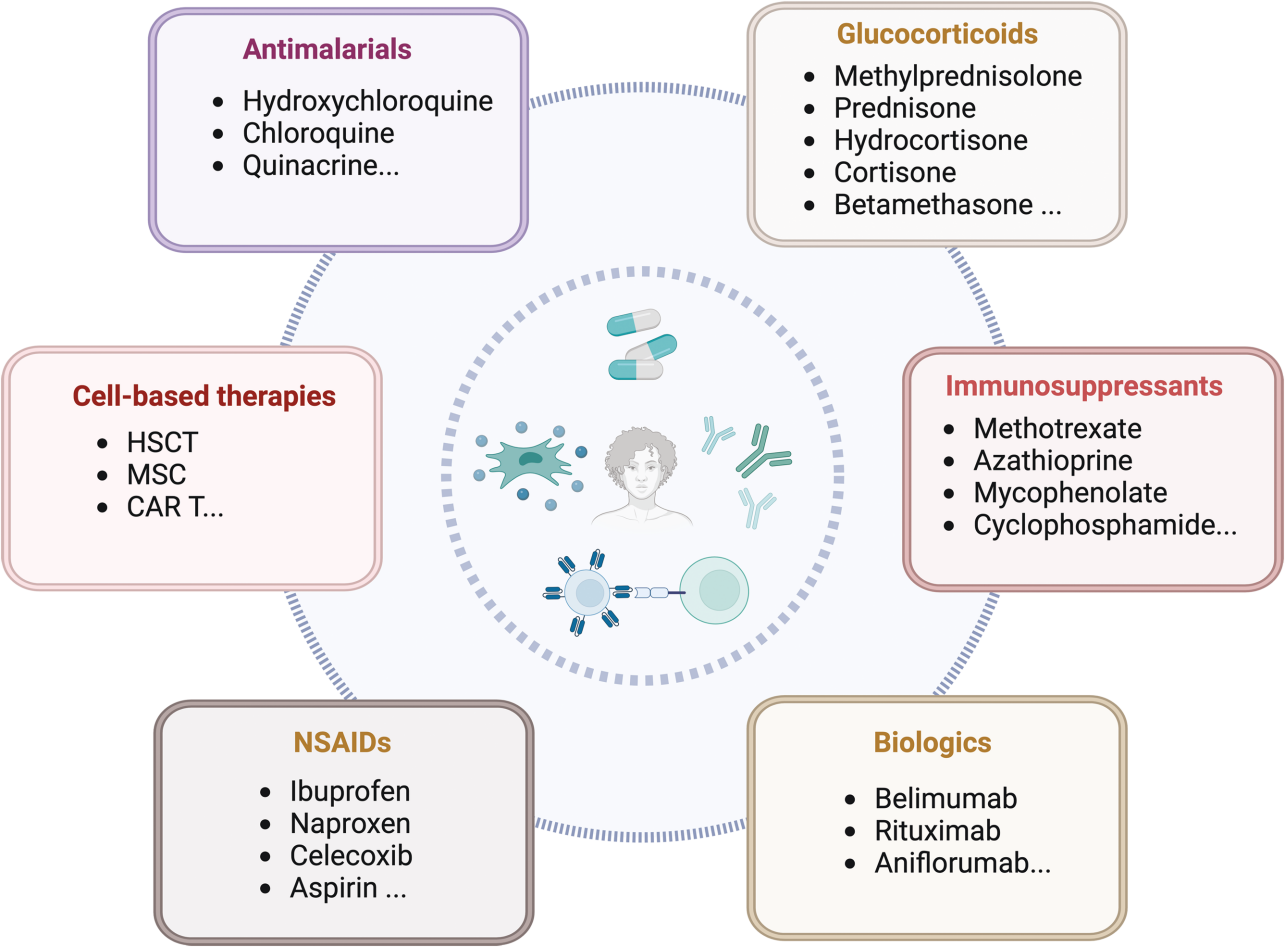
Various treatment strategies for SLE. Abbreviations: CAR T cell, chimeric antigen receptor T cell; HSCT, hematopoietic stem cell transplantation; MSC, mesenchymal stem cell; NSAIDs, nonsteroidal anti-inflammatory drugs. The figure was created in BioRender.com.

SLE presents as a multifaceted condition with diverse phenotypes and clinical symptoms. The broad spectrum of clinical manifestations in SLE is likely due to its molecular diversity and the unpredictability of changes in disease activity. Hence, finding treatment regimens for SLE is still challenging. Well-known organizations such as the Pan American League Against Rheumatism (PANLAR), the European League Against Rheumatism (EULAR), the British Society of Rheumatology (BSR), and the Chinese Rheumatology Association have developed guidelines for the management of SLE.^2,9-11^ Current guidelines recommend the use of antimalarial drugs such as hydroxychloroquine (HCQ), glucocorticoids (GCs), nonsteroidal anti-inflammatory drugs (NSAIDs), and often immunosuppressive therapies such as azathioprine and cyclosporin A for treating persistent disease progression and decreasing GC use (Figure 1). Although these conventional treatments are able to mitigate the symptoms of SLE, they are not curative. In addition, these treatments can lead to significant side effects, such as infections due to their immune suppression effect.^12^ In particular, the use of GCs increases the risk of secondary diabetes, bone disease, metabolic disease, and cardiovascular disease.^13^ Treatment with monoclonal antibodies such as belimumab and anifrolumab (Saphnelo) is likely to reduce disease activity. However, the use of these biological agents is limited to a certain extent because of their high cost and the fact that they are not commonly available in every country. In addition, memory B cells and plasma cells are less responsive to treatment with belimumab.^14^

Notably, even with advances in drug development, a significant portion of patients suffering relapses or they are refractory to conventional treatment.^6^ Therefore, SLE treatment remains challenging for these patients.

## Hematopoietic stem cell transplantation

Hematopoietic stem cells (HSCs) are stem cells that give rise to all types of blood cells. In 1997, HSCs were first used to treat SLE, yielding favorable clinical outcomes.^15^ Since then, numerous clinical studies have been carried out worldwide. In 2022, the European Society for Blood and Marrow Transplantation (EBMT) published a summary of HSCT for treating autoimmune disease. According to this report, 112 patients with SLE were registered at EBMT between 1994 and 2021.^7^

**Figure 2.**
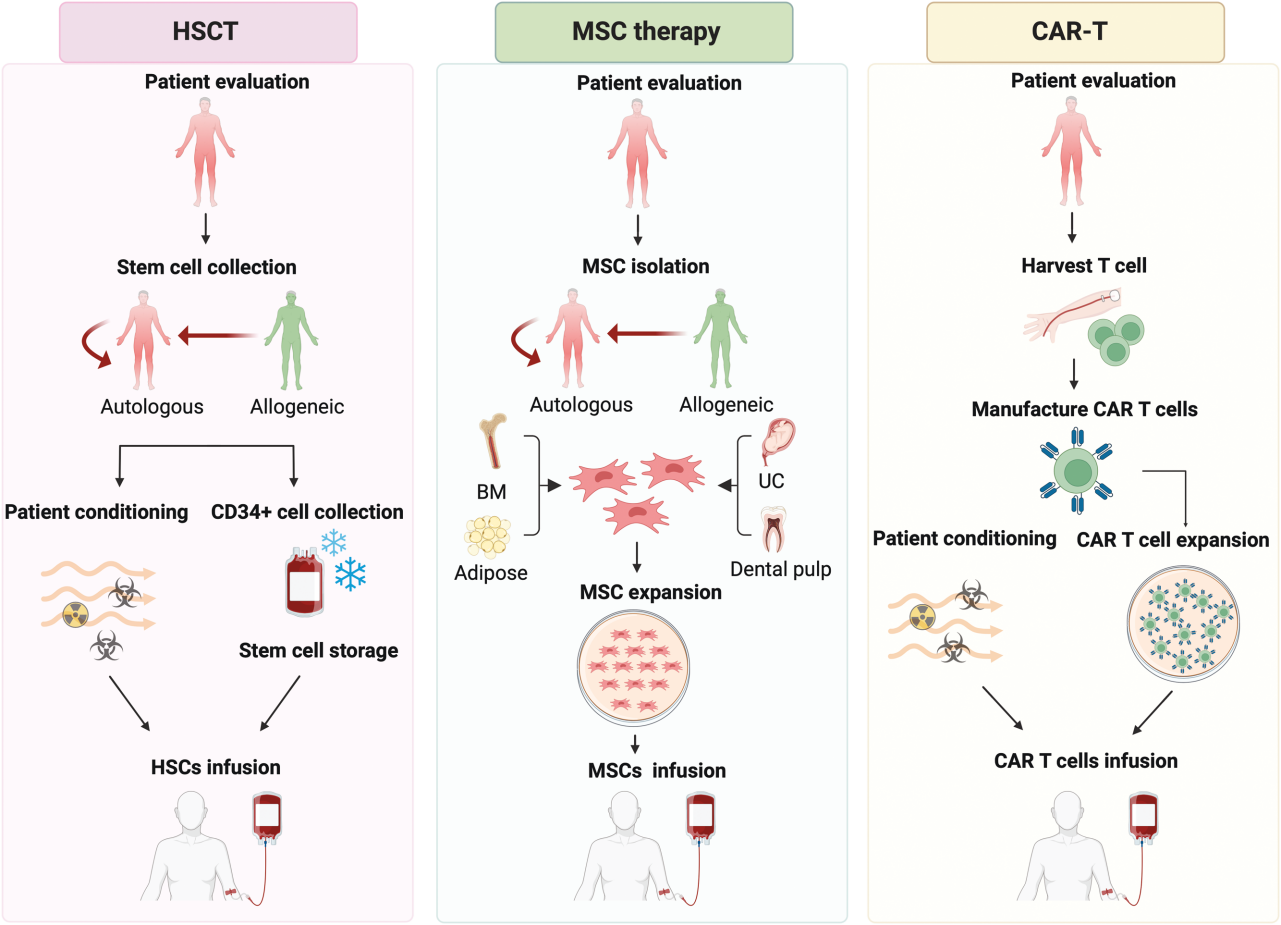
Cell therapies, including hematopoietic stem cell transplantation (HSCT), mesenchymal stem cell (MSC) therapy, and chimeric antigen receptor T (CAR T) therapy, for SLE treatment. Abbreviations: BM: bone marrow; UC: umbilical cord. The figure was created in BioRender.com.

The cells used in HSCT can be sourced from either the patient (autologous) or a donor (allogeneic). In fact, autologous HSCT is generally preferred to allogeneic HSCT due to its more favorable safety profile. The autologous HSCT procedure typically involves the collection of cells from the patient’s peripheral blood, upon pretreatment with granulocyte colony-stimulating factor (G-CSF) either with or without cyclophosphamide. During the mobilization period, CD34 + cells are enriched in peripheral blood and then collected via apheresis. Subsequently, patients undergo chemotherapy and immunosuppression before their enriched CD34 + cell apheresis product is reinfused (Figure 2).

The fundamental idea behind employing HSCT to treat SLE is based on the concept that the conditioning regimen, coupled with the infusion of HSCs, can effectively reset the immune system. This process involves the elimination of autoreactive immune cells and facilitates the regeneration of the hematopoietic and immune system.^16-18^

The largest multicenter trial involving 339 patients revealed a disease-free survival rate of approximately 50%-66% at 5 years despite the discontinuation of immunosuppressive and other targeted disease-modifying therapies.^19^ While most of the studies showed remission, the risk of relapse increased with longer follow-up periods. Although HSCT is a viable option for SLE management, its precise clinical utility needs to be further evaluated in well-designed studies.^20^ Currently, there is one active clinical trial registered on clinicaltrials.gov (NCT05029336) evaluating CD3/CD19-depleted autologous HSCT (Table 1).

**Table 1.** Summary of ongoing clinical trials of cell therapies for the treatment of SLE (clinicaltrials.gov, updated on March 2024, status: active**).**

	Clinical trial No.	Cell source	Phase, N	Study duration	Study design	Country
**HSC transplantation**						
1	NCT05029336	Autologous HSC, CD3/CD19 depletion	Phase II, *N* = 20	2023-2031	Single arm, open label	US
**MSC therapy**						
1	NCT05631717	UC-MSC	Phase 3, *N* = 40	2022-2025	Randomized, open label	China
2	NCT04835883	Allogenic BM-MSC	Phase II, *N* = 10	2019-2026	Single arm, open label	Korea
3	NCT03917797	UC-MSC	Phase II, *N* = 39	2019-2025	Randomized, controlled, quadruple masking	Chile
4	NCT03673748	BM-MSC	Phase II, *N* = 20	2022-2025	Randomized, placebo controlled, double blind	Spain
5	NCT03562065	UC-MSC	Phase I, 2, *N* = 10	2019-2024	Single arm, open label	France
6	NCT02633163	UC-MSC	Phase II, *N* = 81	2018-2023	Randomized, placebo-controlled, double blind	US
**CAR T therapy**						
1	NCT05474885	CD19/BCMA-CAR T	Phase I, *N* = 15	2022-2025	Single arm, open label	China
2	NCT05085418	CD19/BCMA CAR T	Phase I, *N* = 9	2021-2024	Single arm, open label	China
3	NCT05030779	CD19/BCMA CAR T	Early Phase I, *N* = 9	2021-2022	Single arm, open label	China
4	NCT05938725	KYV-101 Anti-CD19 CAR-T cell	Phase I, *N* = 12	2023-2026	Single arm, open label	US
5	NCT05859997	Allogenic Anti-CD19 CAR-T cells (BRL-301)	*N* = 15	2023-2025	Single arm, open label	China
6	NCT06150651	PiggyBac transposon CAR T cells targeting CD19	Phase I, *N* = 6	2023-2025	Single institution	Thailand
7	NCT06106906	CD19 CAR-T	Phase I/2, *N* = 15	2023-2026	Single center	China
8	NCT06106893	CD19 Universal CAR-γδT cells	Phase I/2, *N* = 15	2024-2026	Single center	China
9	NCT06189157	MB-CART19.1	Phase I/2, *N* = 29	2024-2027	Open label, multicenter, interventional single arm	Germany
10	NCT05988216	Universal CAR T Cells (BRL-301)	N = 12	2023-2025	Open label, single group	China
11	NCT06277427	CD19/BCMA CAR T	*N* = 24	2024-2027	Open label, single group	China
12	NCT06056921	CD19 CAR T	Phase I, *N* = 24	2023-2026	Single arm	China
13	NCT05858684	CD19-BCMA CAR T	Early phase I, *N* = 24	2023-2025	Exploratory phase, single arm, nonrandomized, open label	China
14	NCT05798117	CD19 CAR T	Phase I/2, *N* = 24	2023-2026	Open label, multicenter	Switzerland
15	NCT06153095	CD19 CAR T	Phase I/2, *N* = 30	2024-2027	Multicenter, open label	US
16	NCT05765006	CD19 CAR T	Phase I, *N* = 24	2023-2025	Open label, single arm, multicenter	China
17	NCT05930314	CD19 CAR T	Early phase I, *N* = 12	2023-2025	Investigator-initiated, single arm, open label, single dose	China
18	NCT06121297	CD19 CAR T	Phase I/2, *N* = 24	2024-2027	Open label	US
19	NCT05846347	CD19-BCMA CAR T	Phase I, *N* = 15	2023-2025	Single arm, nonrandomized, open label	China
20	NCT06249438	CD20/BCMA-directed CAR-T	Phase I, *N* = 30	2024-2040	Investigator-initiated, multicenter, open label	China
21	NCT05869955	CD19 CAR T	Phase I, *N* = 43	2023-2025	Multicenter, open label	US
22	NCT06038474	CD19-BCMA CAR T	Phase 2, *N* = 30	2024-2027	Open label, single group	US
23	NCT06294236	CD19 CAR T	Phase I, *N* = 36	2024-2027	Nonrandomized, open label	US
24	NCT06285279	CAR T	Phase I, *N* = 24	2024-2028	Single center, open label, dose-escalation	China
25	NCT06222853	CD19 CAR T	Phase I, *N* = 19	2024-2028	Investigator-initiated trial	China
**Other cell therapies**						
1	NCT05566977	Autologous adoptive T regulatory cell	Phase I, *N* = 20, not yet recruiting	2022-2024	Single arm, open label	Egypt
2	NCT06010472	CAR NK cells	Early phase I, *N* = 12	2023-2025	Single arm, open-label pilot	China
3	NCT06255028	CAR-iNK cells	Phase I, *N* = 26	2024-2028	Multicenter, dose finding	US

**Abbreviations:** BCMA, B-cell maturation antigen; BM-MSCs, bone marrow-derived MSCs; CAR T cells, chimeric antigen receptor T cells; CAR-iNK cells, induced pluripotent stem cell-derived CAR-NK; CAR-NK cells, chimeric antigen receptor natural killer cells; CD19, cluster of differentiation 19; HSC, hematopoietic stem cell; HSCT, HSC transplantation; MSC, mesenchymal stem cell; MSCT, MSC transplantation; UC-MSCs, umbilical cord-derived MSCs..

Preliminary results suggest that HSCT holds promise as a therapeutic option for SLE; however, there are still obstacles to overcome toward its more widespread application. First, it has been noted that HSCT is associated with high transplant-related mortality and relapse. In the study of Jayne et al, approximately one-third of patients experienced relapse following HSCT, with a procedure-related mortality rate of 12% in severely ill patients.^21^ Hwang et al used HSCT for the treatment of lupus nephritis and reported that the treatment mortality rate was 5%, the 5-year disease-free survival rate was 53%, and the relapse rate was 27%.^22^ Second, common complications post-HSCT, such as cytomegalovirus (CMV) infection and bacterial/fungal infections, may occur. Other adverse events post-HSCT, including allergies, infections, elevated liver enzymes, bone pain, and heart failure, are relatively frequent. Additionally, long-term complications of HSCT include immune imbalances that may lead to secondary autoimmune diseases.^23,24^ Third, it remains unclear which specific components of the memory compartment require targeting and how extensive lymphocyte lineage depletion should be to achieve sustained responses in patients with SLE.

Due to these considerations, HSCT for SLE is still reserved for patients who have not sufficiently responded to standard therapy. According to the EBMT recommendations, potential candidates for HSCT typically present with sustained or relapsed active British Isles Lupus Assessment Group (BILAG) category A. These candidates remain steroid-dependent despite receiving optimal standard therapy for at least 6 months, which may involve mycophenolate mofetil or cyclophosphamide with or without monoclonal antibodies. Patients must have documented evidence of visceral involvement or refractory SLE.^25^ Current evidence and expert consensus suggest that HSCT for SLE is considered a “clinical option” for patients experiencing active disease despite chronic immunosuppression, with or without B-cell-targeted therapies.

## Mesenchymal stem-cell therapy

MSCs are adult stem cells with the unique capability to self-renew and differentiate into various cell types within the body. MSC therapy has emerged as a treatment option for various autoimmune diseases, such as rheumatoid arthritis, type 1 diabetes, multiple sclerosis, inflammatory bowel disease, Sjogren’s syndrome, and autoimmune liver disease.^26^ MSC therapy has also been explored as a potential treatment for SLE in recent decades.^27-29^ At the time of this review, there were 17 clinical trials on the treatment of SLE with MSCs registered at clinicaltrials.gov, among which 6 clinical studies were active (Table 1).

Bone marrow, umbilical cord, and adipose tissues are commonly used as sources for MSCs. MSC therapy typically involves the isolation of MSCs, followed by cell expansion and infusion into patients. Patients undergoing this therapy do not require chemotherapy before cell infusion (Figure 2). The readily available stem cell sources, low immune rejection characteristics, and no pretreatment chemotherapy requirement confer advantages to this treatment approach. The molecular mechanism of MSCs relies on their ability to regulate both adaptive and innate immune cells.^30^ Thus, the rationale for utilizing MSCs for SLE treatment is to supply large amounts of stem cells to aid the patient’s ability to fight inflammation and alleviate autoimmune symptoms. Although the precise mechanisms through which MSCs exert their immunomodulatory and regenerative effects are not fully understood, it is plausible that multiple mechanisms are involved.^31^

For more than 10 years, the clinical outcomes of patients treated with MSCs have varied across different studies (Supplementary Table S1). The findings from most clinical studies have suggested that MSC therapies are safe, and yielded encouraging results in terms of the amelioration of disease activity. A meta-analysis encompassing 12 studies involving 586 patients revealed that the MSC treatment group experienced a significant reduction in Systemic Lupus Erythematosus Disease Activity Index (SLEDAI) and BILAG scores within 12 months, accompanied by a notable improvement in renal function.^32^ Overall, MSC therapy improves SLE symptoms; however, it does not constitute a complete curative option. Since further confirmation of the effectiveness of MSC therapy is still needed, this therapy is currently used in treating patients with refractory SLE and is rarely used in patients with mild SLE.

Notably, allogeneic MSCs are more commonly used than autologous MSCs in the treatment of SLE. Evidence suggests that autologous MSCs are not beneficial for patients with SLE, implying the abnormalities of MSCs themselves in the progression of SLE.^33-35^ In contrast to MSCs derived from healthy donors, MSCs obtained from patients with SLE exhibit immunomodulatory deficiencies and a morphological bias toward senescent cells.^35^ In a study involving 2 SLE patients infused with autologous bone marrow-derived MSCs, disease activity indices, including BILAG and SLEDAI, remained largely unchanged, despite an increase in circulating Treg cell counts during a 14-week follow-up period.^33^

Various limitations hinder the widespread adoptation of MSCs in clinical treatment. First, MSCs can be obtained from various tissue sources from different donors via different methods and can be cultured through various protocols, resulting in heterogeneous cell populations. This makes it difficult to clearly identify general efficacy or to establish benchmarks for the use of MSCs. Establishing a standard protocols for the preparation of homogenous MSC products is crucial for limiting the variation in the MSC population and ensuring consistent therapeutic effects. Second, graft rejection in MSC therapy has also been considered by researchers. While allogenic MSC therapy is considered suitable for treating autoimmune diseases, study finding have raised concerns about graft rejection following repeated infusions.^36,37^ Third, there are currently no studies comparing different MSC sources for SLE treatment. Consequently, the selection of a suitable stem cell source for SLE treatment is still based on the experience of the investigators.

### Engineered MSCs

MSCs can be genetically engineered to enhance their immune regulatory functions in the treatment of SLE. A study by Xu et al showed that genetically modified MSCs overexpressing IL-37 significantly reduced inflammation, reduced the levels of serological autoantibodies, improved disease syndromes, and prolonged the survival of SLE mice.^38^ Other strategies aim to increase the in vivo survival of MSCs. The encapsulation of MSCs within microgels and microparticles could protect MSCs from host immune system attacks while preserving their secretory and immunomodulatory properties. These interventions have shown promise in suppressing disease progression in lupus mice.^39,40^ However, these approaches have not yet been tested in human trials.

### MSC-derived extracellular vesicles

Like other living cells, MSCs secrete various types of vesicles into the extracellular environment. These extracellular vesicles (EVs) contain many biological materials that play important roles in cell-to-cell communication and participate in numerous physiological and pathological processes.^41^

Although MSC therapies have shown promise for treating SLE, the function and mechanism of MSC-derived extracellular vesicles (MSC-EVs) remain unclear. Research findings suggest that MSC-EVs have biological effects similar to those of their parent cells.^42^ Studies in animal models have shown that both human umbilical cord mesenchymal stem cells (hUCMSCs) and hUCMSC-derived EVs inhibit CD4+ T cells, promote the production of T helper (Th)17 cells, and increase the secretion of interleukin (IL)-17 and transforming growth factor beta 1 (TGF-β1) when cocultured with activated splenic mononuclear cells extracted from MRL/lpr mice.^43^ While only hUCMSCs affected CD19+ B cells and the production of interferon-gamma (IFN-γ) and IL-4, hUCMSC-derived EVs partially contributed to the immunoregulatory effects of hUCMSCs in SLE. Moreover, hUCMSC-derived exosomes exhibited anti-inflammatory effects by favoring CD206+ M2 and CD163+ M2 cells over CD68+ M1 and HLA-DR+ M1 cells in vitro. The administration of exosomes derived from hUCMSCs to MRL/lpr mice resulted in greater infiltration of CD14+ CD163+ M2 cells and Treg cells than of CD14+ CD11c+ M1 cells into the spleen in the hUCMSC-treated group than in the phosphate-buffered saline (PBS)-treated group. As a result of these anti-inflammatory and immunomodulatory effects, the survival rate of experimental mice increased, and nephritis and liver and lung injuries diminished.^44^ Similarly, exosomes from bone marrow-derived mesenchymal stem cells (BM-MSCs) have been shown to promote the polarization of macrophages toward a specific anti-inflammatory phenotype and Treg-cell recruitment.^45^

The potential use of MSC-EVs for the treatment of SLE has been explored. The success of preclinical studies of MSC-EVs for SLE treatment shows the great promise of MSC-EVs for future therapy; however, no clinical trials utilizing MSC-EVs have been conducted in SLE patients thus far.

## Chimeric antigen receptor T (CAR T)-cell therapy

Over the past few years, CAR T-cell therapy has demonstrated remarkable therapeutic efficacy in certain types of hematological malignancies.^46,47^ The first CAR T-cell product, Kymriah, engineered for treating B-cell acute lymphoblastic leukemia (ALL) and B-cell non-Hodgkin lymphoma (NHL), received approval in 2017. Presently, dozens of CAR T-cell therapies for hematologic malignancies have been approved by the Food and Drug Administration (FDA).

CAR T-cell therapy involves the genetic modification of T cells to target particular cells. The procedure encompasses the collection of the patient’s T cells through apheresis, genetic engineering, and ex vivo expansion, followed by reinfusion into patients (Figure 2).

The promising outcomes of CAR T-cell therapies in treating B-cell malignancies has sparked broader investigations into their potential application in treating autoimmune conditions, such as SLE.^48^ In individuals with SLE, B cells are responsible for the production of autoantibodies, suggesting that targeting B cells could be a viable treatment approach for alleviating B-cell-mediated autoimmunity. Numerous clinical trials have been conducted investigating the safety and efficacy of CAR T-cell therapy for lupus (Table 1). A research team in Germany documented a case where a CD19 CAR T-cell product was administered to eliminate B cells in a patient with SLE.^49^ Following this, the researchers extended their investigation to 5 patients, noting a remission of SLE clinical symptoms in all patients within 3 months. Crucially, drug-free remission was sustained for up to 12 months.^8^

While preliminary data show promise, several factors are hindering the widespread adoption of CAR T-cell therapies for SLE treatment. First, patients undergoing this treatment typically require 3-5 days of chemotherapy to deplete their immune cells prior to CAR T-cell infusion. The lymphodepletion procedure increases susceptibility to infections and other side effects. Second, there are concerns regarding the risk of common side effects following CAR T-cell infusion, such as cytokine release syndrome (CRS) and neurotoxicity. Third, the expense of treatment remains excessively high. Additionally, despite promising initial results, the number of studies and treated patients remains limited, and the long-term follow-up duration has not been thoroughly assessed. CAR T cells represent a promising approach for treating SLE with encouraging preliminary results; still, the effectiveness of these treatments needs to be further evaluated in future studies with larger cohort sizes and long-term follow-up assessments.

## Regulatory T cells (Tregs) therapy

Treg cells are a specialized type of CD4+ T cells that play an essential role in the establishment and maintenance of immune tolerance. Studies have reported a decrease in the number of CD4+ CD25+ FOXP3+ Treg cells and impaired Treg-cell-mediated suppression in individuals with SLE, and this decrease is inversely correlated with the severity of the disease.^50-53^ Hence, modulating both the quantity and functionality of Treg cells has emerged as a promising therapeutic option for managing SLE. Several strategies have been developed to enhance the Treg cell response, including the use of immunosuppressive drugs such as rapamycin/sirolimus^54^ or the administration of low-dose IL-2 to increase the number of circulating Treg cells and their proliferative potential.^55^

Cell therapy based on in vitro-expanded Treg cells has shown that adoptive Tregs transplantation might have beneficial effects on the management of SLE. The case of the first patient treated with autologous adoptive Treg cells was reported in the study of Dall’Era et al^56^ autologous CD4+ CD127^lo^CD25^hi^ Treg cells were isolated from one SLE patient, expanded in vitro, and subsequently infused at a dose of 1 × 10^8^ cells. The research group reported that Treg infusion led to an increase in activated Treg activity and altered T helper (Th) activity from a Th1 to a Th17 cell response at the site of inflammation, which could impact the clinical manifestations of the disease. Apart from this study, there have been no other reported results from clinical trials. Currently, there is only one (01) ongoing clinical trial registered on clinicaltrials.gov (NCT05566977).

It is important to highlight several drawbacks of unmodified Treg cells, notably their rarity and the challenges associated with culturing them in vitro.^57^ Moreover, these cells are polyclonal and target multiple antigens. Hence, engineered Treg cells equipped with antigen-specific Treg cells, such as CARs, direct these cells toward specific autoantigens and incorporate costimulatory domains to promote their expansion.^58^ In this strategy, a CAR construct recognizing specific autoantigens is introduced to generate CAR Treg cells. Thus far, no CAR Treg cells have been specifically used in patients with SLE. One challenge could be the identification of a unique antigen for autoreactive B and T cells, as many potential autoantigens may contribute to the pathology of this disease. Furthermore, Treg-cell-derived exosomes with their immunosuppressive effects may become a novel research direction in SLE management.^59^

## Natural killer cell therapy

NK cells are innate lymphocytes that are crucial for immune surveillance. Studies have reported dysfunction in NK cells among patients with SLE, characterized by decreased cell numbers and cytotoxicity, as well as impaired cytokine production and antibody-dependent cellular cytotoxicity.^60,61^ Humbel et al demonstrated that NK cells from patients with SLE exhibited increased CD38 expression but failed to adequately upregulate SLAMF1 and SLAMF7 upon cytokine stimulation.^61^ The engagement of SLAMF7 and/or CD38 with specific monoclonal antibodies, such as elotuzumab and/or daratumumab, respectively, promoted NK-cell degranulation, cytokine production, and cytotoxicity, enabling the elimination of cells secreting autoreactive antibodies. These findings suggest that restoring NK-cell function could ameliorate SLE symptoms.

NK cells can be engineered to target specific cells. For instance, engineered NK cells with CARs specifically target cells expressing high levels of human programmed cell death protein 1 (PD-1) were shown to eliminate follicular helper T cells (T_FH_ cells).^62^ This study demonstrated that CAR-NK cells could effectively eliminate T_FH_ cells, thereby inhibiting B-cell proliferation and antibody production in vitro and in a humanized mouse model of lupus-like disease. Importantly, this targeted approach did not adversely affect Treg cells or memory T cells. These findings suggest that directing therapeutic interventions at T_FH_ cells may be a promising strategy for the treatment of lupus in the future. Recently, a research group in China has conducted a phase I clinical trial (NCT06010472) to evaluate the safety and efficacy of CD19-targeted CAR-NK cells (KN5501) in patients with moderate to severe refractory SLE. Another research group in the US investigating induced pluripotent stem cell (iPSC)-derived NK cells with CD19-directed CARs has treated participants in a phase I clinical trial named Calipso-1 (NCT06255028). These clinical trials are in progress, and no results have been reported thus far.

## Other potential cell therapies

Numerous cell therapy approaches have been studied in vitro and in animal models, with encouraging results. In the subsequent section, we will delve into the well-studied cell therapies that show promise for clinical application in humans.

### Regulatory B cells

Regulatory B cells (Breg cells) constitute a specific subset of B cells that negatively regulate immune responses through the secretion of immunosuppressive cytokines.^63^ Given the excessive immune activation characteristic of SLE, the immunosuppressive properties of Breg cells have potential for ameliorating the symptoms of this disease. Studies have reported functional impairment of Breg cells in SLE patients.^64^ Therefore, restoring the functionality of Breg cells may be a strategy for ameliorating the symptoms of SLE. This therapeutic approach involves extracting Breg cells from the patient’s peripheral blood, activating and expanding these cells ex vivo, and subsequently infusing them back into the patient.

Findings from mouse model studies have suggested that transplanting Breg cells could alleviate disease symptoms. For example, transplanting Breg cells (B10 cells) from wild-type mice into lupus model mice significantly extended their survival.^65^ Another study showed that in vitro transplanting of anti-CD40-generated T2 B cells (T2-like Breg cells) significantly alleviated renal disease and increased survival through an IL-10-dependent mechanism.^66^ Nevertheless, significant uncertainties persist regarding Breg cells, such as identifying specific markers for their precise identification and how to maintain their functional stability in vivo. Further investigations should prioritize addressing these unknowns for a better understanding of Breg cells and their practical application in SLE treatment.

### Myeloid-derived suppressor cells

Myeloid-derived suppressor cells (MDSCs) are a heterogeneous population of cells originating from the myeloid lineage. There are 2 major subsets of MDSCs: granulocytic/polymorphonuclear MDSCs (PMN-MDSCs) and monocytic MDSCs (M-MDSCs).^67^ The primary attribute of MDSCs lies in their capacity to suppress immune responses, which include those orchestrated by T cells, B cells, and NK cells. Studies have indicated a significant increase in the number of MDSCs in SLE patients, which is positively correlated with disease severity.^68-70^ The adoptive transplantation of MDSCs has demonstrated efficacy in alleviating autoimmune diseases.^71^ The reported beneficial effects of adoptive M-MDSC transplantation were an alleviation of SLE symptoms in a pristane-induced lupus mouse model.^72^ Similarly, the in vitro transfer of MDSCs from healthy mice into a sanroque mouse model of SLE-induced Breg cell expansion via inducible nitric oxide synthase (iNOS), resulting in the amelioration of autoimmunity.^73^ Interestingly, MDSC-derived EVs have shown therapeutic effects in modulating immune responses.^74^ These findings indicate promising potential therapeutic strategies for SLE utilizing MDSC-based therapies. However, challenges related to the in vitro production of MDSCs and the clinical cost need to be considered when applying MDSC-based immunotherapy for SLE.^75,76^

### Dendritic cells

Dendritic cells (DCs) are a heterogeneous group of bone marrow-derived cells that play important roles in immunosurveillance, antigen presentation, and tolerance. The involvement of DCs in the pathogenesis of SLE is well recognized.^23^ Tolerogenic (immature) DCs or monocytes (cells able to differentiate into DCs) can promote T-cell hyporesponsiveness and foster immune tolerance. Therefore, tolerogenic DCs or monocytes have emerged as attractive therapeutic targets for SLE.^77^ Preclinical studies involving DC immunotherapy in mice have been reported. The administration of autologous tolerogenic DCs derived from the bone marrow of MRL-Fas^lpr^ and NZM2410 model mice alleviated certain SLE symptoms and reduced nuclear antibody titers, but no significant improvements in proteinuria or glomerulonephritis were observed.^78^ In a human trial, Jonny et al reported significant improvements in disease manifestations in a pediatric patient with SLE following autologous DC transplantation.^79^

Several clinical trials have explored DC immunotherapy for autoimmune diseases such as rheumatoid arthritis, type I diabetes mellitus, multiple sclerosis, and Crohn’s disease.^80-83^ However, only a limited number of studies have investigated autologous DC immunotherapy for SLE, likely due to the challenges associated with identifying SLE-specific antigens and the impaired functions of DCs in SLE patients.^84,85^

### Innate lymphoid cells

Innate lymphoid cells (ILCs) are a heterogeneous group of immune cells characterized by antigen-independent activation. ILCs are important effectors of innate immunity because of their rapid production of proinflammatory and regulatory cytokines.^86^ In the past decade, increasing attention has been given to the role of innate immune cells and molecules in facilitating and exacerbating SLE. Studies have indicated an altered distribution of circulating ILC subsets in SLE patients.^87,88^ Given their recognized involvement in autoimmune disease pathogenesis, researchers have suggested the potential of ILCs as therapeutic targets.^89^ Evidence from preclinical and experimental animal models suggests the participation of ILCs in various autoimmune diseases, including SLE.^90^ However, further investigation is necessary before considering ILCs as candidates for cell therapy in SLE.

### Monocytes

Monocytes constitute a cell-population derived from hematopoietic myeloid precursors that play essential roles in immunoregulation and the production of several inflammatory cytokines. The involvement of monocytes in the pathogenesis of SLE is well documented.^91^ Studies have revealed that monocytes excessively produce B-lymphocyte stimulator (BLyS), which promotes the survival and proliferation of B cells.^92^ In murine models, the inhibition of monocyte activation, differentiation, and migration has resulted in therapeutic effects.^93^ These findings suggest that targeting monocytes could be an alternative treatment strategy. A pilot study involving the depletion of monocytes and neutrophils demonstrated clinical improvements in SLE patients.^94^ Further studies on therapies based on monocytes will be instrumental in elucidating their potential value in treating SLE.

### Induced pluripotent stem cell-based therapy

Induced pluripotent stem cells (iPSCs) are stem cells generated from somatic cells that can differentiate into various cell types. iPSCs offer the advantage of being an autologous cell source, thereby circumventing issues related to immune rejection. In the context of SLE, iPSCs derived from patient samples provide a promising platform for pathological studies of disease pathology and drug discovery. Several research groups have successfully generated iPSCs from SLE patients, and these cells have been used to analyze SLE-specific features in iPSC-based studies or have been in vitro differentiated into desired cells, such as CD123+ DCs and hematopoietic and mesenchymal lineage cells.^95-97^ These findings support the potential of iPSC-based methods for both autologous and allogenic cell-replacement therapy in SLE. However, extensive studies are required to advance iPSC-based cell therapy to human clinical trials.

## Selection of cell-therapy approaches for SLE

Among the different cell-therapy approaches that have been applied in human trials, there is a paucity of research data on Treg- and NK-cell therapies, making it premature to assess their effectiveness. In contrast, autologous HSCT and MSC therapies have been used in treating SLE patients, and proof of concept for achieving long-term remission or even drug-free remission has been demonstrated. A new approach to CAR T-cell therapy has been attempted in a small number of patients; however, this approach has great potential in the treatment of the disease. Currently, cell therapy is typically reserved for patients unresponsive to standard treatments or to those in advanced stages of the disease with refractory responses to other therapies. The choice of the suitable cell therapies for an individual depends on various factors and necessitates careful consideration of the benefits and risks (Table 2).

**Table 2.** Comparison of autologous HSC, allogeneic MSC, and CAR T-cell therapy in SLE treatment.

	Autologous HSC therapy	Allogenic MSC therapy	CAR T therapy
Patient recommendation	Patients who do not sufficiently respond to standard therapies^98,99^	Can be administered at different stages of SLE, both early and late stages^27^	Recommended for late-stage patients who do not respond to any other treatment option^8^
Eligible patient	No obvious active infection, no major hematological abnormalities, no active cancers and adequate organ function	No obvious active infection, adequate organ function	No obvious active infection; no prior organ transplantation, adequate organ function, and expected survival period at least 3 months
Rationale of using	Reset immune system^16,18^	Immunomodulatory^31,100^	Reset the antibody-producing lineages^101^
Procedure	Complicated procedure. Requires experienced centres for stem cell collection, processing, administration and patient management post-HSCT due to the complexity of cell engraftment. Requires patient conditioning before cell infusion.	Less complicated procedure. Easily accessible stem cell resource, stem cell processing, administration and patient management post-transplantation. No patient conditioning required	Highly complicated procedure. Requires dedicated equipment and significant technical expertise. CAR T-cell production and administration are time-consuming. Close patient management post-transplantation is required due to the complexity of cell engraftment. Requires patient conditioning.
Side effects	Infections, allergies, organ failure, secondary autoimmune diseases	Mild side effects such as dizziness, headaches, nausea/vomiting, fever	Cytokine release syndrome, neurotoxicity
Safety	Lower	High	Potentially high
Therapeutic effects	Patients achieved complete or partial remission. The rate varied among studies (Supplementary Table S1)	Patients achieved complete or partial remission. The rate varies between studies (Supplementary Table S1)	All patients achieved remission- tentatively, due to small the sample size (Supplementary Table S1)
Relapse rate	Relapse rate is nearly one-third^22^	Relapse rate is between 17% and 23%^102,103^	No relapse observed in the study period^8,101,104^
Cost	Relatively high cost for complicated patient conditioning before transplantation and high-dose immunosuppressive drugs	Medium cost. MSCs have low immunogenicity; therefore, no immunosuppressive drugs are needed	Extremely high cost for complicated CAR T production procedure, patient conditioning and post therapy follow-up

In general, the HSCT procedure is relatively complicated and involves patient conditioning and post-transplantation management due to cell engraftment complexity. However, HSCT does not require cell culture facilities. Although the safety and effectiveness of HSCT have been demonstrated, this therapy is also associated with many risks. The most concerning risk is infection during the conditioning stage and engraftment period. Moreover, HSCT has numerous contraindications depending on the patient’s health status.

The safety and efficacy of MSC transplantation for treating lupus have been reported in several studies.^102,105-107^ Unlike HSCT, the infusion of MSCs entails fewer risks and contraindications, as patients do not need conditioning before cell infusion. Although the production cost of MSCs is still high, the overall cost is favorable compared to that of HSCT due to the shorter hospital stay.

CAR T-cell therapy is the most expensive -cell therapy approach, primarily due to its highly complex procedure, which requires dedicated equipment, substantial technical expertise, and meticulous patient management post-transplantation. As the number of patients treated with CAR T-cell therapy for SLE remains limited, it is still premature to draw conclusions on its safety and effectiveness.

## Conclusion

Despite significant progress in drug development, including the recent approval of monoclonal antibodies, challenges persist in managing SLE. Over the past several decades, -cell therapy approaches have garnered increasing attention for their potential effectiveness in treating SLE. Several noteworthy points include the following:

- Cell therapies such as HSCT and MSC and CAR T-cell therapies are recommended for patients who do not respond to standard treatments or who are refractory to all available treatment options.
- Although HSCT has been used for several decades, the number of treated patients remains limited. Other cell therapies are still in the early stages of clinical application. In this context, it will be of interest to follow the outcomes of ongoing clinical trials.
- Despite the promising results observed, patients need to carefully consider various factors before opting for cell therapy, including safety, therapeutic effects, potential side effects, treatment costs, and overall medical conditions. Therefore, thorough discussions between clinical doctors/specialists and the patient’s family are essential before proceeding with cell therapy.

An objective of ongoing and upcoming human trials is to comprehensively assess the prolonged safety and efficacy of cell therapies. This involves the refinement of techniques to enable better outcomes, long-term remission achievement, and increased disease-free ratio. Additionally, reducing costs could substantially broaden the scope of cell therapy applications for managing SLE.

## Supplementary material

Supplementary material is available at *Stem Cells Translational Medicine* online.

szae044_suppl_Supplementary_Material

## Data Availability

No new data were generated or analyzed in support of this research. None declared.
