# Supplementary material for: Current cell therapies for systemic lupus erythematosus
Source: Stem Cells Transl Med. 2024 Jun 26;13(9):859–72. doi: 10.1093/stcltm/szae044 (PMC11386214; doi:10.1093/stcltm/szae044)
Supplement: szae044_suppl_Supplementary_Material [file szae044_suppl_supplementary_material.docx]

**Supplement table 1: Summary of published clinical studies of cell therapies for SLE treatment**

| **Study** | **Study design, n** | **Patient condition** | **Follow-up** | **Cell source** | **Cell dose** | **Outcomes** | **References** |
| --- | --- | --- | --- | --- | --- | --- | --- |
| **HSC Therapy** | | | | | | | |
| 1 | Single arm, open label,  n = 22 | Refractory LN | Median of 72 (60–80) months | Autologous HSC | 7.3±3.8 × 10^6^ CD34+ cells/kg | 5% (1) treatment-related mortality, 82% (18) patients achieved completed remission, 5% (1) patient achieved partial remission, and 5% (1) patient had no response. The 5-year overall survival and disease-free survival rates were 91% and 53%, respectively. Relapse rate was 27%. The major complications were fever and gastrointestinal tract symptoms. | ^1^ |
| 2 | Single arm, open label  n = 30 | Refractory SLE | 5 years | Autologous HSC |  | No treatment related deaths. No remission in group of 4 patients received a conditioning regimen of cyclophosphamide and alemtuzumab. In the group of 26 patients treated with cyclophosphamide, rATG, and rituximab, disease remission was 92% at 6 months, 92% at one year, 81% at 2 years, 71% at 3 years, and 62% at 4 and 5 years post-HSCT. Improved SLEDAI score, C3 complement, C4 complement, ANA, anti-dsDNA, SF-36 scale. | ^2^ |
| 3 | Single arm, controlled  n = 50 (11 HSCT, 39 non-HSCT) | Refractory SLE | 6 years for 2 cases | Autologous HSC |  | Compare the pregnacy complication rate and fetal outcome in SLE patients treated with autologous HSCT (transplanted group) vs traditional immunosuppressive therapy (non-transplant group). Compare with non-transplant group, the rate of maternal hypertension and lupus nephritis was greatly reduced, the outcome of lupus flare activity of the mother after delivery is significantly improved in the transplanted group. Two patients experienced >6 years of remission post-HSCT. | ^3^ |
| 4 | Single arm, open label  n = 7 | Refractory SLE | 8 years | Autologous HSC | 2 × 10^6^– 6.1 x 10^6^ CD34+ cells/kg | Clinical remissions were observed; anti–dsDNA antibodies were largely disappeared. Autoreactive immunologic memory is depleted and a tolerant, “juvenile” adaptive immune system was developed. Increased naive CD31+CD45RA+CD4+ T cells. 1 case got relapse. | ^4^ |
| 5 | Single arm  n = 8 | SLE | 10 years | Autologous HSC | ≥ 2 × 10^6^ CD34+ cells/kg | Well tolerated HSCT in all patients. Two relapses, which responded to Rituximab. The cumulative SLEDAI score dropped from 90 to 9. | ^5^ |
| 6 | Single arm,  n = 50 (2 died after mobilization, 48 underwent HSCT) | Refractory SLE | Mean 29 months (range, 6 months to 7.5 years) | Autologous HSC, nonmyeloablative | > 1.4 x 10^6^ CD34+ cells/kg | 4% treatment-related mortality. The 5-year survival was 84%, 5-year probability of disease-free survival was 50%. Stabilization of renal function. Stable renal function. Significant improvement in SLEDAI score, ANA, anti-dsDNA, complement, and carbon monoxide diffusion lung capacity adjusted for hemoglobin. | ^6^ |
| 7 | Single arm,  n = 15 | Severe and Refractory Lupus | Median 36 months (range 12–66 months). | Autologous HSC | > 1.4 x 10^6^ CD34+ cells/kg | No deaths occurred. The SLEDAI score has declined to ≤  5 in 12 patients. Complement and anti–dsDNA levels have normalized and marked improvements in end organ function have occurred in all subjects. Of the 12 patients followed up for >1 year after HSCT, 10 have discontinued immunosuppressive medications, and tapered the prednisone dosage. 2 patients had clinical evidence of recurrence of active lupus. | ^7^ |
| 8 | Single arm,  n = 9 (2 were excluded, 7 underwent HSCT) | Severe SLE | Median 25 months (range 12–40 months) | Autologous HSC | The median infused CD34 dose was 2.4 x 10^6^ cells/kg | All patients were free from signs of active lupus. Renal, cardiac, pulmonary, and serological markers, and T-cell phenotype and repertoire had normalized. | ^8^ |
| 9 | n = 1 | SLE | 12 months | Autologous HSC | ≥ 2 × 10^6^ CD34+ cells/kg | Patient entered a persisted complete clinical and serological remission during  > 1 year of follow-up | ^9^ |
| 10 | n = 2 | SLE | 1st patient: 12 months  2nd patient: 6 months | Autologous HSC | ≥ 2 × 10^6^ CD34+ cells/kg | Both patients have had no evidence of active disease since transplantation. SLEDAI score markedly decreased from 32 and 37 to 0. For the 1st patient, renal function has stabilized. For the first time since disease onset, C3, C4 and ANA were normal with the patient off all immunosuppressive medications, including corticosteroids. For the 2nd patient, hemoptysis and pulmonary infiltrates have resolved and corticosteroids has been gradually tapered. | ^10^ |
| 11 | n = 1 | SLE | 6 months | Autologous HSC |  | Complete clinical and serologic remission, despite the gradual discontinuation of immunosuppressive medications, including corticosteroids. | ^11^ |
| **MSC Therapy** | | | | | | | |
| 1 | Uncontrolled, phase I,  n = 6 | Refractory SLE | 24 weeks | UC-MSC | 1 x 10^6^ cells/kg | 5/6 patients achieved SRI of 4. Significant reduction in CD27IgD double negative B cells, switched memory B cells and activated naïve B cells, with increased transitional B cells in the five patients who met the end points | ^12^ |
| 2 | open-label, single-arm, phase I,  n = 7 | SLE | 28 days | Haploidentical allogeneic BM-MSC (CS20AT04) | starting dose is 2×10^6^ cells/kg or escalated dose of 3×10^6^ cells/kg | No DLT at both initial dose and escalated dose. Three AEs (diarrhea, toothache, and arthralgia) were reported. The maximum tolerated dose was determined to be 3.0×10^6 cells/kg in patients with LN. | ^13^ |
| 3 | Phase I,  n = 9 | Refractory LN | 12 months | Allogeneic AD-MSC | 2 x 10^6^ cells/kg | Median score of SLEDAI decreased significantly from 16 to 6 after sixth months. Urine protein levels significantly decreased during the first month and remained lower than baseline up to the 3rd month. Complete renal response and partial response were observed in 33.3% and 44.4% of the patients, respectively during the first 3 months post-intervention. | ^14^ |
| 4 | Retrospective cohort study,  n = 69 | SLE | 12 months | Allogeneic BM- or UC-MSC | 1 or 2 doses, 1 x 10^6^ cells/kg | 58% achieved low disease activity (LDA) and 23% achieved complete remission (CR). The SLEDAI score reduce to 5 after 12 months. Achieving CR was associated with older age, no arthralgia/arthritis at baseline, and no prior use of CYC or HCQ. | ^15^ |
| 5 | Phase I,II,  n=21 | SLE | Max 6 months | UC-MSC | 1 x 10^6^ cells/kg | No advese event during 24h and 72h after infusion. SLEDAI score remarkably declined after 1 month and 6 months. | ^16^ |
| 6 | Open-label, phase II,  n = 81 | Severe and refractory SLE | Mean 6 years (5 - 8 years) | Allogeneic BM- and/or UC-MSC | Dose vary, 1 x 10^6^ cells/kg | 5-year overall survival rate was 84%, remission rate was 34% (27% CCR, 7% PCR), relapsed was 24%. SLEDAI score, serum albumin, complement C3, peripheral white blood cell, and platelet numbers, proteinuria levels, continued to improve during the follow-up. | ^17^ |
| 7 | n =3 | SLE | 9 months | Allogenic MSC | 90 x 10^6^ cells/patient | SLEDAI scores revealed substantial remissions in two patients and partial for the third patient | ^18^ |
| 8 | Randomized double-blind, placebo-controlled, n = 18 (12 hUC-MSC and 6 placebo) | LN | 12 months | Allogeneic UC-MSC | Two doses of 1 x 10^8^ cells with 7-day interval | CR rate and improvements in serum albumin, complement, renal function, SLEDAI and BILAG scores were similar in both groups.  The trial showed no apparent treatment effect of UC-MSC compare to control. | ^19^ |
| 9 | Multicenter,  n = 40 | Active and refractory SLE | 12 months | Allogeneic UC-MSC | Two doses, 1 x 10^6^ cells on day 0 and day 7 | MSCT was well tolerated, and no transplantation related AEs were observed. The overall survival rate was 92.5%. 32.5% and 27.5%, achieved major clinical response and partial clinical response, respectively. 9-month and 12-month relapse was 12.5% and 16.7%, respectively. SLEDAI & BILAG scores, 24-hour proteinuria decreased. | ^20^ |
| 10 | Open-label and single-center,  n = 81 | Active and refractory LN | 12 months | Allogeneic BM- or UC-MSC | 1 dose, 1 x10^6^ cells/kg | 12-month overall survival rate of was 95 %; 60.5 % achieved renal remission, 22.4 % had renal flare. BILAG & SLEDAI scores declined, GFR improved. No TAEs were observed. | ^21^ |
| 11 | n = 35 | Cytopenia refractory SLE | Average 21 months (range 6–45 months) | Allogeneic  BM- and/or UC-MSC | Dose vary, 1 x 10^6^ cells/kg | Significant improvements in blood cell count. Disease activity declined. CR was accompanied by increased Treg and decreased Th17. No TAEs were observed. | ^22^ |
| 12 | n = 87 | Severe and refractory SLE | Mean follow-up period of 27 months (range 12–48 months) | Allogeneic BM- or UC-MSC | 1×10^6^ cells/kg | The overall survival rate was 94% with 23% relapse. CCR rate was 28% at 1 year, 31% at 2 years, 42% at 3 years, and 50% at 4 years. SLEDAI score, levels of serum autoantibodies, albumin, and complements declined. No TAEs were observed. | ^23^ |
| 13 | n = 58 (30 received single MSCT, 28 received double MSCT) | Refractory SLE | Mean 27 months (12-48 months) in single transplantation group  Mean 26 months (12-40 months) in double transplantation group. | Allogeneic BM-MSCs or UC-MSC | Single MSCT:  1 dose, 1 x 10^6^ cells/kg  Double MSCT:  2 doses of 1 x 10^6^ cells/kg | Survival rate was 100% for single and 96.4% for double transplantation group. Rate of complete remission during 4 years follow-up was 53.3% in single and 28.6% in double transplantation. Disease relapse rate was 26.7% for single and 22.2% for double transplantation | ^24^ |
| 14 | Single arm, n =16 | Refractory SLE | The median follow-up time was 8.25 months (range 3–28 months) | UC-MSC | 1 dose, 1 x 10^6^ cells/kg, IV | MSC infusion was safe and well tolerated. Improvement in SLEDAI score and renal function in all patients. No treatment-related deaths. No relapse observed. | ^25^ |
| 15 | n = 15 | Refractory SLE | 17.2±9.5 months | Allogeneic BM-MSC | 1 dose, 1 x10^6^ cells/kg | Marked decrease in the SLEDAI score and 24 h proteinuria; anti-dsDNA levels decreased; non-renal-related manifestations improved significantly. No serious AEs were reported. | ^26^ |
| 16 | n = 2 | SLE | 14 weeks | Autologous BM-MSC | 1 dose, 1 x 10^6^ cells/kg | No adverse effects or change in disease activity indexes were observed, y in spite of increasing Treg cell counts. | ^27^ |
| **CAR T Therapy** | | | | | | | |
| 1 | n = 5 | Refractory SLE | 5-17 months | Autologous CD19 CAR-T cell | 1 dose, 1x10^6^ cells/kg | CAR T expand rapidly in vivo got peak on average at day 9. CAR T cell treatment was well tolerated with only mild CRS. All five patients achieved remission according to DORIS criteria and SLEDAI score (decrease to 0) after 3 months. Drug-free remission was maintained during longer follow-up (median (range) of 8 (12) months) and even after the reappearance of B cells. Reappearing B cells were naive and showed non-class-switched B cell receptors. | ^28^ |
| 2 | n = 1 | Severe and refractory SLE presented with active LN | 44 days | Autologous CD19 CAR-T cell | 1 dose, 1.1×10^6^ cells/kg | No AEs related to CAR T-cell therapy was observed. CAR T-cell numbers rapidly increased and be detectable during the next 7 weeks. Expansion of CAR T cells preceded the complete and sustained depletion of circulating B cells. dsDNA level autoantibodies decreased rapidly within 5 weeks, and the low C3 and C4 levels normalized. These signs of serologic remission were paralleled by clinical remission with proteinuria decreasing. SLEDAI score decreased from 16 to 0. | ^29^ |
| 3 | n = 1 | SLE with stage IV diffuse large B-cell lymphoma (DLBCL) | 37 weeks | CD19-BCMA cCAR T cell | 5.3 × 10^6^ cells/kg | CAR T cells expanded in vivo with the first peak at 7 days after infusion and was seen within two months. B-cells remained at undetectable levels until day 198 post cCAR, with recovery to normal levels 9 months post-cCAR. Plasma cells in the marrow were depleted. Despite the discontinuation of prednisone, C3 and C4 levels remained within normal limits. ANA titers remained undetectable after 37 weeks. Total immunoglobulin levels marked reduced. | ^30^ |
| **Other cell therapies** | | | | | | | |
| 1 | N=1 | SLE | 48-week | Autologous regulatory T Cells | 1 × 10^8^ Tregs | Treg infusion increased activated Treg activity and altered Th1 toward Th17 response in inflammation | ^31^ |

*Abbreviations: SLE: systemic lupus erythematosus: LN: lupus*nephritis; *MSC: Mesenchymal stem cell; MSCT: MSC transplantation; BM-MSCs: bone marrow-derived MSCs; UC-MSCs: umbilical cord-derived MSCs; AD-MSCs: adipose tissue-derived MSCs; HSC: hematopoietic stem cell; HSCT: HSC transplantation; CAR: chimeric antigen receptor; SLEDAI: Systemic Lupus Erythematosus Disease Activity Index; BILAG: British Isles Lupus Assessment Group; CR: clinical remission; CCR: complete clinical remission; PCR: partial clinical remission; CFR: Glomerular filtration rate; PGA: patient global assessment; LDA: low disease activity; MCTD: mixed connective tissue disease; JIA: juvenile idiopathic arthritis; DLT: dose-limiting toxicity; AE: adverse event; TAE: transplantation-related AE:; CYC: Cyclophosphamide; HCQ: Hydroxychloroquine; WBC: white blood cell; CRS: cytokine-release syndrome; Tregs: Regulatory T cells; DORIS: Definitions Of Remission In SLE, cCAR:* Compound CAR*; DLBCL: Diffuse large B cell lymphoma; SRI:* *Systemic Lupus Erythematosus Responder Index; ANA:* *anti-nuclear antibody.*

**REFERENCES**

1. Huang X, Chen W, Ren G, et al. Autologous Hematopoietic Stem Cell Transplantation for Refractory Lupus Nephritis. *Clin J Am Soc Nephrol*. 2019;14(5):719-727. doi:10.2215/CJN.10570918

2. Burt RK, Han X, Gozdziak P, et al. Five year follow-up after autologous peripheral blood hematopoietic stem cell transplantation for refractory, chronic, corticosteroid-dependent systemic lupus erythematosus: effect of conditioning regimen on outcome. *Bone Marrow Transplant*. 2018;53(6):692-700. doi:10.1038/s41409-018-0173-x

3. Meng J, Wang J, Liang W, Qin S, Wu C. Long-term remission after successful pregnancy in autologous peripheral blood stem cell transplanted system lupus erythematosus patients. *Rheumatol Int*. 2011;31(5):691-694. doi:10.1007/s00296-010-1588-x

4. Alexander T, Thiel A, Rosen O, et al. Depletion of autoreactive immunologic memory followed by autologous hematopoietic stem cell transplantation in patients with refractory SLE induces long-term remission through de novo generation of a juvenile and tolerant immune system. *Blood*. 2009;113(1):214-223. doi:10.1182/blood-2008-07-168286

5. Gualandi F, Bruno B, Van LINT M t., et al. Autologous Stem Cell Transplantation for Severe Autoimmune Diseases. *Annals of the New York Academy of Sciences*. 2007;1110(1):455-464. doi:10.1196/annals.1423.048

6. Burt RK, Traynor A, Statkute L, et al. Nonmyeloablative hematopoietic stem cell transplantation for systemic lupus erythematosus. *JAMA*. 2006;295(5):527-535. doi:10.1001/jama.295.5.527

7. Traynor AE, Barr WG, Rosa RM, et al. Hematopoietic stem cell transplantation for severe and refractory lupus. Analysis after five years and fifteen patients. *Arthritis Rheum*. 2002;46(11):2917-2923. doi:10.1002/art.10594

8. Traynor AE, Schroeder J, Rosa RM, et al. Treatment of severe systemic lupus erythematosus with high-dose chemotherapy and haemopoietic stem-cell transplantation: a phase I study. *Lancet*. 2000;356(9231):701-707. doi:10.1016/S0140-6736(00)02627-1

9. Traynor A, Burt RK. Haematopoietic stem cell transplantation for active systemic lupus erythematosus. *Rheumatology*. 1999;38(8):767-772. doi:10.1093/rheumatology/38.8.767

10. Burt RK, Traynor AE, Pope R, et al. Treatment of Autoimmune Disease by Intense Immunosuppressive Conditioning and Autologous Hematopoietic Stem Cell Transplantation. *Blood*. 1998;92(10):3505-3514. doi:10.1182/blood.V92.10.3505

11. Burt RK, Traynor A, Ramsey-Goldman R. Hematopoietic Stem-Cell Transplantation for Systemic Lupus Erythematosus. *N Engl J Med*. 1997;337(24):1777-1778. doi:10.1056/NEJM199712113372416

12. Kamen DL, Wallace C, Li Z, et al. Safety, immunological effects and clinical response in a phase I trial of umbilical cord mesenchymal stromal cells in patients with treatment refractory SLE. *Lupus Science & Medicine*. 2022;9(1):e000704. doi:10.1136/lupus-2022-000704

13. Chun S, Choi CB, Kim MS, et al. Safety and tolerability of bone marrow-derived mesenchymal stem cells in lupus animal models and a phase I clinical trial in humans. *Lupus*. 2022;31(10):1245-1253. doi:10.1177/09612033221111957

14. Ranjbar A, Hassanzadeh H, Jahandoust F, et al. Allogeneic adipose-derived mesenchymal stromal cell transplantation for refractory lupus nephritis: Results of a phase I clinical trial. *Current Research in Translational Medicine*. 2022;70(2):103324. doi:10.1016/j.retram.2021.103324

15. Wen L, Labopin M, Badoglio M, Wang D, Sun L, Farge-Bancel D. Prognostic Factors for Clinical Response in Systemic Lupus Erythematosus Patients Treated by Allogeneic Mesenchymal Stem Cells. *Stem Cells Int*. 2019;2019:7061408. doi:10.1155/2019/7061408

16. Yuan X, Qin X, Wang D, et al. Mesenchymal stem cell therapy induces FLT3L and CD1c+ dendritic cells in systemic lupus erythematosus patients. *Nat Commun*. 2019;10(1):2498. doi:10.1038/s41467-019-10491-8

17. Wang D, Zhang H, Liang J, et al. A Long-Term Follow-Up Study of Allogeneic Mesenchymal Stem/Stromal Cell Transplantation in Patients with Drug-Resistant Systemic Lupus Erythematosus. *Stem Cell Reports*. 2018;10(3):933-941. doi:10.1016/j.stemcr.2018.01.029

18. Barbado J, Tabera S, Sánchez A, García-Sancho J. Therapeutic potential of allogeneic mesenchymal stromal cells transplantation for lupus nephritis. *Lupus*. 2018;27(13):2161-2165. doi:10.1177/0961203318804922

19. Deng D, Zhang P, Guo Y, Lim TO. A randomised double-blind, placebo-controlled trial of allogeneic umbilical cord-derived mesenchymal stem cell for lupus nephritis. *Ann Rheum Dis*. 2017;76(8):1436-1439. doi:10.1136/annrheumdis-2017-211073

20. Wang D, Li J, Zhang Y, et al. Umbilical cord mesenchymal stem cell transplantation in active and refractory systemic lupus erythematosus: a multicenter clinical study. *Arthritis Res Ther*. 2014;16(2):R79. doi:10.1186/ar4520

21. Gu F, Wang D, Zhang H, et al. Allogeneic mesenchymal stem cell transplantation for lupus nephritis patients refractory to conventional therapy. *Clin Rheumatol*. 2014;33(11):1611-1619. doi:10.1007/s10067-014-2754-4

22. Li X, Wang D, Liang J, Zhang H, Sun L. Mesenchymal SCT ameliorates refractory cytopenia in patients with systemic lupus erythematosus. *Bone Marrow Transplant*. 2013;48(4):544-550. doi:10.1038/bmt.2012.184

23. Wang D, Zhang H, Liang J, et al. Allogeneic Mesenchymal Stem Cell Transplantation in Severe and Refractory Systemic Lupus Erythematosus: 4 Years of Experience. *Cell Transplant*. 2013;22(12):2267-2277. doi:10.3727/096368911X582769c

24. Wang D, Akiyama K, Zhang H, et al. Double allogenic mesenchymal stem cells transplantations could not enhance therapeutic effect compared with single transplantation in systemic lupus erythematosus. *Clin Dev Immunol*. 2012;2012:273291. doi:10.1155/2012/273291

25. Sun L, Wang D, Liang J, et al. Umbilical cord mesenchymal stem cell transplantation in severe and refractory systemic lupus erythematosus. *Arthritis Rheum*. 2010;62(8):2467-2475. doi:10.1002/art.27548

26. Liang J, Zhang H, Hua B, et al. Allogenic mesenchymal stem cells transplantation in refractory systemic lupus erythematosus: a pilot clinical study. *Annals of the Rheumatic Diseases*. 2010;69(8):1423-1429. doi:10.1136/ard.2009.123463

27. Carrion F, Nova E, Ruiz C, et al. Autologous mesenchymal stem cell treatment increased T regulatory cells with no effect on disease activity in two systemic lupus erythematosus patients. *Lupus*. 2010;19(3):317-322. doi:10.1177/0961203309348983

28. Mackensen A, Müller F, Mougiakakos D, et al. Anti-CD19 CAR T cell therapy for refractory systemic lupus erythematosus. *Nat Med*. 2022;28(10):2124-2132. doi:10.1038/s41591-022-02017-5

29. Mougiakakos D, Krönke G, Völkl S, et al. CD19-Targeted CAR T Cells in Refractory Systemic Lupus Erythematosus. *New England Journal of Medicine*. 2021;385(6):567-569. doi:10.1056/NEJMc2107725

30. Zhang W, Feng J, Cinquina A, et al. Treatment of Systemic Lupus Erythematosus using BCMA-CD19 Compound CAR. *Stem Cell Rev Rep*. 2021;17(6):2120-2123. doi:10.1007/s12015-021-10251-6

31. Dall’Era M, Pauli ML, Remedios K, et al. Adoptive Treg Cell Therapy in a Patient With Systemic Lupus Erythematosus. *Arthritis Rheumatol*. 2019;71(3):431-440. doi:10.1002/art.40737
